# Exploring Indirect Sources of Human Exposure to Perfluoroalkyl Carboxylates (PFCAs): Evaluating Uptake, Elimination, and Biotransformation of Polyfluoroalkyl Phosphate Esters (PAPs) in the Rat

**DOI:** 10.1289/ehp.1002409

**Published:** 2010-11-08

**Authors:** Jessica C. D’eon, Scott A. Mabury

**Affiliations:** Department of Chemistry, University of Toronto, Toronto, Ontario, Canada

**Keywords:** biotransformation, human exposure, perfluorinated carboxylates, perfluorooctanoic acid, pharmacokinetics, polyfluoroalkyl phosphate esters

## Abstract

**Background:**

Perfluorinated carboxylic acids (PFCAs) are ubiquitous in human sera worldwide. Biotransformation of the polyfluoroalkyl phosphate esters (PAPs) is a possible source of PFCA exposure, because PAPs are used in food-contact paper packaging and have been observed in human sera.

**Objectives:**

We determined pharmacokinetic parameters for the PAP monoesters (monoPAPs) and PAP diesters (diPAPs), as well as biotransformation yields to the PFCAs, using a rat model.

**Methods:**

The animals were dosed intravenously or by oral gavage with a mixture of 4:2, 6:2, 8:2, and 10:2 monoPAP or diPAP chain lengths. Concentrations of the PAPs and PFCAs, as well as metabolic intermediates and phase II metabolites, were monitored over time in blood, urine, and feces.

**Results:**

The diPAPs were bioavailable, with bioavailability decreasing as the chain length increased from 4 to 10 perfluorinated carbons. The monoPAPs were not absorbed from the gut; however, we found evidence to suggest phosphate-ester cleavage within the gut contents. We observed biotransformation to the PFCAs for both monoPAP and diPAP congeners.

**Conclusions:**

Using experimentally derived biotransformation yields, perfluorooctanoic acid (PFOA) sera concentrations were predicted from the biotransformation of 8:2 diPAP at concentrations observed in human serum. Because of the long human serum half-life of PFOA, biotransformation of diPAP even with low-level exposure could over time result in significant exposure to PFOA. Although humans are exposed directly to PFCAs in food and dust, the pharmacokinetic parameters determined here suggest that PAP exposure should be considered a significant indirect source of human PFCA contamination.

Perfluorinated carboxylates (PFCAs) and perfluorinated sulfonates (PFSAs) have been detected in human sera worldwide (Calafat et al. 2007; Haug et al. 2009; Kärrman et al. 2006; Olsen et al. 2008). PFCA and PFSA human contamination are surprisingly similar in North America (Calafat et al. 2007; Olsen et al. 2008), Europe (Haug et al. 2009), and Australia (Kärrman et al. 2006), given the marked differences in environmental contamination (Yamashita et al. 2005). Despite the ubiquity of PFCAs and PFSAs in human sera, major sources of this contamination are not well understood.

Fluorochemicals have historically been manufactured via two different processes. Electrochemical fluorination (ECF) was used in the production of perfluorooctane sulfonyl fluoride [POSF; F(CF_2_)_8_SO_2_F], which was further functionalized into the sulfonamido ethanols. Telomerization was used in the production of the fluorotelomer alcohols [FTOHs; F(CF_2_)*_x_*CH_2_CH_2_OH]. These two alcohols were then used to manufacture commercial surfactants and polymers. A major difference between these processes is that telomerization produces exclusively the linear isomer, whereas ECF produces fluorochemicals with a mixture of structural isomers (Kissa 2002).

A major shift in industrial fluorochemical production occurred in 2000, when 3M, the major manufacturer of the POSF-based chemistries, began phasing out its perfluorooctyl materials (3M 2000). This change in production was mirrored in human sera, as concentrations of perfluorooctane sulfonate (PFOS) and perfluorooctanoate (PFOA) began to decrease around the year 2000 (Calafat et al. 2007; Haug et al. 2009; Olsen et al. 2008). These studies generally show a larger decrease in concentrations of PFOS compared with PFOA. PFOS has a longer human serum elimination half-life (Olsen et al. 2007), thus suggesting continued exposure to PFOA without similar exposure to PFOS.

Consistent with continued exposure to PFOA, concentrations of the longer chain PFCAs—perfluorononanoate (PFNA), perfluorodecanoate (PFDA), and perfluoroundecanoate (PFUnA)—have continued to increase in human sera since the 2000 POSF phase-out (Calafat et al. 2007; Haug et al. 2009). The most plausible explanation for continued PFCA exposure, without continued exposure to PFOS, is human exposure to fluorotelomer-based commercial materials.

Exposure to perfluorinated acids from the biotransformation of precursor materials is not a new idea. The sulfonamide-based materials can be biotransformed to PFOS (Xu et al. 2004), and acetate metabolites have frequently been detected in human sera (Calafat et al. 2007; Haug et al. 2009; Olsen et al. 2008). Biotransformation from FTOH to PFCA has previously been observed in a rat model (Fasano et al. 2006; Hagen et al. 1981). Fluorotelomer-based materials are composed of a mixture of chain lengths (Kissa 2002), so biotransformation of these compounds could explain concurrent human exposure to PFCAs of several chain lengths. In addition, the PFCA isomer profile in human sera is suggestive of a fluorotelomer source, because it is almost entirely linear (De Silva and Mabury 2006), with a diminished contribution from specific ECF isomers found to be more bioaccumulative than the linear isomer (Benskin et al. 2009; De Silva et al. 2009).

One class of fluorotelomer-based commercial products with a high potential for human exposure is the polyfluoroalkyl phosphate esters (PAPs). Commercial PAP formulations contain a mixture of fluorinated chain lengths as well as phosphate mono-, di-, and triesters (Begley et al. 2008; Kissa 2002). By synthesizing individual congeners in-house, we simplified this complex mixture to four PAP diesters [diPAPs; *x*:2, (OH)P(O)(OCH_2_CH_2_(CF_2_)*_x_*F)_2_, where *x* = 4, 6, 8, or 10] and four PAP monoesters [monoPAPs; *x*:2, (OH)_2_P(O)(OCH_2_CH_2_(CF_2_)*_x_*F), where *x* = 4, 6, 8, or 10]. PAPs are used to greaseproof food-contact paper products and have a demonstrated ability to migrate from packaging into food (Begley et al. 2008). Biotransformation from PAP to PFCA has previously been observed in rats (D’eon and Mabury 2007) and in a microbial system (Lee et al. 2010). Human PAP exposure has also been confirmed by the detection of several diPAP congeners in human sera at microgram per liter concentrations (D’eon et al. 2009). The explicit hypothesis of this work is that PAP biotransformation contributes to the PFCA burden observed in human sera. We tested this hypothesis by determining PAP uptake, elimination, and biotransformation parameters to properly constrain PFCA production from PAP exposure.

## Materials and Methods

### Chemical purchase, synthesis, and purity

PAPs were synthesized as described previously (D’eon and Mabury 2007), and diPAPs were purified by preparatory liquid chromatography. We stripped monoPAPs of FTOHs by repeat dissolution in acetone, followed by solvent evaporation under nitrogen [for details, see Supplemental Material (doi:10.1289/ehp.1002409)].

### Animal treatment

This research was conducted under an animal use protocol approved by the University of Toronto Animal Care Committee. All animals were treated humanely and with regard for alleviation of suffering. Sprague-Dawley rats were obtained from Charles River Laboratories Inc. (Sherbrooke, QC, Canada) The animals were singly housed and exposed to a 12/12 hr light/dark cycle with food and water available *ad libitum*. Blood samples were collected from the saphenous vein using Microvette CB 300 vials (Sarstedt AG & Co., Montreal, QC, Canada) embedded with lithium-heparin as an anticoagulant. Whole-blood samples were stored at −20°C, and urine and feces samples were stored at 4°C.

### Uptake experiment

Two groups of four 8-week-old male rats were administered a mixture of 4:2, 6:2, 8:2, and 10:2 diPAP or monoPAP, respectively, each at about 50 mg/kg, at 4 mL/kg by oral gavage without prior fasting. Because the diPAPs have demonstrated solubility in emulsions (Begley et al. 2008), the dosing vehicle used was 50:50 propylene glycol:water, with 0.1% soy lecithin added as an emulsifier. Two control male rats were administered clean vehicle at 4 mL/kg [for details, see Supplemental Material, Table 3 (doi:10.1289/ehp.1002409)].

### Elimination experiment

Two groups of four 8-week-old male rats were administered a mixture of 4:2, 6:2, 8:2, and 10:2 diPAP or monoPAP, respectively, each at about 15 mg/kg, at 2 mL/kg by intravenous (IV) injection into the lateral tail vein. The dosing vehicle was 50:50 propylene glycol:water. Two control male rats were administered clean vehicle at 2 mL/kg [for details see Supplemental Material, Table 3 (doi:10.1289/ehp.1002409)].

### Blood collection

Blood was collected from all animals 3 days before dosing to establish background contamination. For the uptake experiment, blood samples were collected at 1, 3, and 6 hr and 1, 2, 4, 7, 10, 14, 18, and 22 days after dosing. For the elimination experiment, blood samples were collected at 2, 4, and 6 hr and 1, 2, 4, 8, 11, 15, and 18 days after dosing. On the final sampling day, the animals were sacrificed by carbon dioxide asphyxiation, and about 10 mL of blood was collected by cardiac puncture into a BD Vacutainer embedded with lithium-heparin.

### Urine and feces collection

For both experiments the animals were placed in metabolic cages for 48 hr postdosing, with urine and feces samples collected when available at 3, 6, 9, 24, 34, and 48 hr postdosing.

### Toxic end points

No toxicity data are available in the open scientific literature for the PAPs. However, the PAP dosing concentrations used here were consistent with previous investigations (D’eon and Mabury 2007), where no toxicity was observed. One fatality occurred in the diPAP dose group during blood sampling for the uptake experiment, for unknown reasons. Two fatalities occurred during dose administration in the elimination experiment (one animal from each dose group). Complications related to the use of propylene glycol as a dosing vehicle was the suspected cause of the fatalities. No physical signs of stress were observed in the other animals at any time during either study.

### Analytical details

The compounds of interest fall into four categories: *a*) the diPAP and monoPAP parent compounds, *b*) the saturated and unsaturated fluorotelomer carboxylic acid (FTCA and FTUCA) metabolic intermediates, *c*) the FTOH-sulfate and FTOH-glucuronide phase II metabolites, and *d*) the final PFCA biotransformation products [see Supplemental Material, Table 1 (doi:10.1289/ehp.1002409)]. We analyzed these compounds in whole blood, urine, and feces samples. For full analytical details, see Supplemental Material. All PFCAs, FTCAs, FTUCAs, and phase II metabolites were quantified by internal calibration. Because internal standards were not available, monoPAPs and diPAPs were quantified by standard addition. We performed spike and recovery experments to validate the extraction and quantification techniques used (for details, see Supplemental Material).

### Data analysis

Analyte and matrix specific limits of detection (LODs) and limits of quantification (LOQs) are provided in Supplemental Material (doi:10.1289/ehp.1002409). For the purpose of calculating means, values < LOD were assigned a value of zero, and values < LOQ but > LOD were used unaltered but are indicated by asterisks in figures and parentheses in tables. Values are presented using the arithmetic mean ± SE. Full data sets are available in Supplemental Material.

We included one procedural blank with the extraction of each time point. The only analyte observed in the procedural blanks or the control animals was PFOA; however, the observed contamination was consistently < LOQ and considered negligible.

For details regarding the calculation of pharmacokinetic parameters, see Supplemental Material (doi:10.1289/ehp.1002409).

**Figure 1:**
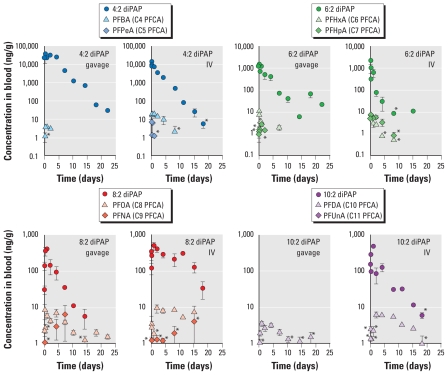
Arithmetic mean ± SE for the concentrations of the diPAP parent compounds and their expected primary PFCA biotransformation products observed in rats (*n* = 3) after diPAP dose administered either IV or via oral gavage. Values < LOD were assigned a value of zero; values < LOQ but > LOD are indicated by an asterisk [for full data sets, see Supplemental Material (doi:10.1289/ehp.1002409)]. Note that the diPAPs were dosed as a mixture; for clarity, the PFCA biotransformation products are plotted together with the expected parent diPAP.

Despite the rigorous purity analyses performed, the diPAP IV dose was inadvertently contaminated with perfluorohexanoic acid (PFHxA). Because of the short serum half-life of PFHxA in male rats, this contamination was quickly eliminated (Ohmori et al. 2003). For clarity, the first three PFHxA time points (2–6 hr) from the diPAP IV experiment are not included in Figure 1. For full PFHxA concentrations from this experiment, see Supplemental Material (doi:10.1289/ehp.1002409).

In the present study and our previous investigations, diPAP identification has involved two multiple reaction monitoring (MRM) transitions, one to the monoPAP and the other to [H_2_PO_4_]^−^ (97 *m/z*) (D’eon et al. 2009; D’eon and Mabury 2007). In our previous biotransformation study (D’eon and Mabury 2007), only the MRM transition to [H_2_PO_4_]^−^ was monitored for 8:2 monoPAP. From an in-house synthesized 8:2 FTOH-sulfate standard, we recognized that the MRM transition of this species was indistinguishable from that of 8:2 monoPAP (543 *m/z* > 97 *m/z*). This potential interference was resolved in the present study by adding an additional MRM transition to [PO_3_]^−^ (79 *m/z*) for monoPAP quantification, as well as chromatographically separating the FTOH-sulfates and monoPAPs.

## Results

Absorption of 8:2 monoPAP and 8:2 diPAP, as well as biotransformation to PFOA, has previously been observed in rats (D’eon and Mabury 2007). Our hypothesis that PAPs may contribute to the burden of PFCAs observed in human sera was supported by the observation of diPAPs at microgram per liter concentrations in human sera from the United States (D’eon et al. 2009). To properly constrain PFCA production from PAP exposure, in the present study we undertook an in-depth evaluation of PAP uptake, elimination, and biotransformation.

### Uptake

We calculated percent bioavailability using the area under the curve after gavage and IV dose administration (AUC_gavage_ and AUC_iv_, respectively), normalized to the concentration of the respective doses:


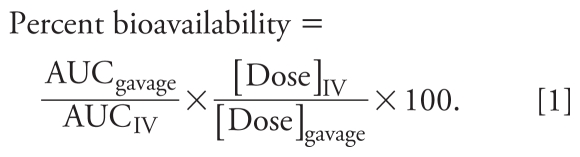


Figure 1 shows diPAP concentration–time plots after gavage and IV dosing. Bioavailabilities determined using Equation 1 were 190%, 74%, and 5% for 4:2, 6:2, and 8:2 diPAP congeners, respectively (10:2 diPAP was < LOD in the gavage experiment). Because a bioavailability > 100% is not possible, diPAP bioavailability may be overestimated.

We collected the first blood sample 2 hr after IV dose administration; in retrospect, there could have been an initial excretion phase that was not properly captured. Conversely, toxicological complications with the IV dose could also be responsible for this apparent discrepancy. In the absence of any confounding factors, it is possible that there was a physiological cause for the percent bioavailability > 100%. Ward et al. (2004) observed the same phenomenon for the drug SB-265123, an organic carboxylate, and implicated saturable intestinal secretory transport proteins in the overestimation of the compound’s bioavailability. Fecal excretion after diPAP IV dosing was greatest for 6:2 diPAP (see “Fecal elimination”), a trend that may suggest active elimination. Organic anion transport proteins have been implicated in the renal control of the PFCAs (Andersen et al. 2008) and may also be involved in the uptake of PFOA into rat hepatocytes (Han et al. 2008). Therefore, it is possible that similar transport proteins may be involved in the movement of fluorinated acids, including the diPAPs, between the gut contents and the bloodstream.

We observed no monoPAP congeners in the blood of the dosed animals in the present study. Because this study was designed to investigate PAP kinetics in the blood, it is not possible to determine if the monoPAPs partitioned into other compartments within the body. Further mass balance studies are warranted to fully characterize the fate of these chemicals in the body.

The lack of monoPAPs we observed in the blood contrasts results of a previous study (D’eon and Mabury 2007), in which we observed 8:2 monoPAP concentrations > 100 ng/g after oral gavage at 200 mg/kg. As described in “Material and Methods,” it is possible that in the previous study we overestimated the oral absorption of 8:2 monoPAP because of interference from the phase II metabolite 8:2 FTOH-sulfate.

### Elimination kinetics in the blood

We determined elimination half-lives from whole blood for the diPAP congeners after both the gavage and IV dosing (Table 1). Half-lives after IV dosing (mean ± SE) were 1.6 ± 0.1, 2.1 ± 0.3, 4.8 ± 1.0, and 3.3 ± 0.4 days for 4:2, 6:2, 8:2, and 10:2 diPAP, respectively. Half-lives after oral gavage dosing were 2.0 ± 0.1, 3.9 ± 0.7, and 2.4 ± 0.2 days for 4:2, 6:2, and 8:2 diPAP, respectively. We did not observe 10:2 diPAP in the blood of the animals from the gavage experiment, so no half-life could be calculated. As expected, the diPAP half-lives generally increased with increasing diPAP chain length.

Half-lives were recently determined for the di-substituted perfluoroalkyl phosphinic acids (diPFPAs) in both male and female rats (D’eon and Mabury 2010). The diPFPAs are structurally similar to the diPAPs, except their alkyl chains are fully fluorinated and bound directly to the phosphorus center. Half-lives of the diPFPAs in male rats after intraperitoneal injection ranged from 1.8 to 5.4 days. These half-lives are similar to those observed here for the diPAPs.

### Renal elimination

We did not observe the monoPAPs in urine after IV or gavage dosing. This may result from low renal elimination or their inability to reach the kidneys through the blood.

The only diPAP congener we observed in urine was 4:2 diPAP. However, renal elimination is unlikely to be the major route of 4:2 diPAP elimination because the levels observed could account for only 0.03% of the administered gavage dose, and we detected 4:2 diPAP only sporadically in urine samples after IV dosing. This result is consistent with the low renal elimination observed for the longer chain PFCA, PFDA (Ohmori et al. 2003).

The shorter chain PFCAs (≤ C8), as well as 4:2 FTOH-glucuronide and 4:2 FTOH-sulfate, were consistently observed in the urine samples from all dosing regimes (for full data sets, see Supplemental Material, Tables 25–30 (doi:10.1289/ehp.1002409)].

### Fecal elimination

We did not observe the monoPAPs in fecal samples collected after IV dosing. This may result from low monoPAP fecal elimination or their partitioning to tissues other than the liver, where biliary excretion is possible. Conversely, 6:2, 8:2, and 10:2 monoPAPs were present in feces collected 24 hr after gavage dosing at levels that account for about 1% of the administered gavage dose. [We encountered analytical challenges with the extraction and analysis of 4:2 monoPAP in the fecal samples; for details, see Supplemental Material (doi:10.1289/ehp.1002409).] Because the monoPAPs present in feces after gavage dosing likely resulted from residual dose, the low concentrations observed suggest that the monoPAPs either are taken up by the gut or are transformed within the gut contents.

We observed diPAPs in fecal samples after both IV and gavage dosing, so delineating residual dose from contributions from fecal excretion was not possible. Forty-eight hours after IV dosing, fecal elimination could account for 3%, 9%, 1%, and 0.04% of 4:2, 6:2, 8:2, and 10:2 diPAP, respectively; however, 48 hr after gavage dosing, fecal elimination was higher: 21%, 65%, and 34% for 4:2, 6:2, and 8:2 diPAP, respectively.

We detected no biotransformation products in the feces [for full data sets, see Supplemental Material, Table 31 (doi:10.1289/ehp.1002409)].

### Biotransformation

**Figure 2:**
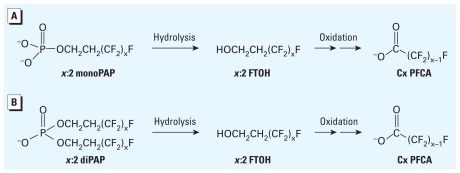
Biotransformation of *x*:2 monoPAP (*A*) and *x*:2 diPAP (*B*).

As reported previously in rats (D’eon and Mabury 2007) and in a microbial system (Lee et al. 2010), PAP biotransformation is expected to proceed via enzyme-mediated hydrolysis of the phosphate ester linkage, yielding the corresponding FTOH, which is then available for oxidation (Figure 2).

The β-oxidation product is the major PFCA produced from FTOH oxidation (i.e., 8:2 FTOH to PFOA), with minor production of the α-oxidation product (i.e., 8:2 FTOH to PFNA) and shorter chain PFCAs (Fasano et al. 2006; Hagen et al. 1981). The concentration of PFCA products we observed in the blood of dosed animals is dependent on both biotransformation yields and PFCA pharmacokinetics. Generally, the PFCA congener profile present in the blood of both the diPAP- and monoPAP-dosed animals agreed with the expected biotransformation pathway, with elevated levels of the even-chain-length PFCAs compared with the odd-chain-length congeners [Table 1; for full data sets, see Supplemental Material (doi:10.1289/ehp.1002409)]. Concentrations of the PFCA products observed in the blood of the diPAP-dosed animals are plotted with their expected diPAP parent in Figure 1. Although we did not observe monoPAPs in the blood of the dosed animals, we did observe PFCA biotransformation products after monoPAP dosing (Table 1).

We also monitored metabolic intermediates and phase II metabolites in the blood of the dosed animals [for full data sets, see Supplemental Material (doi:10.1289/ehp.1002409)]. The highest concentrations of metabolic intermediates and phase II metabolites were observed after monoPAP oral gavage. We frequently detected 6:2 FTCA, 6:2 FTUCA, 8:2 FTCA, and 8:2 FTUCA in blood samples taken 2, 4, and 6 hr postdosing. Both 5:3 and 7:3 FTCA were present in all of the samples from 2 hr to 2 days postdosing. The 4:2 and 6:2 FTOH-glucuronides were observed at 2 hr and 6 hr postdosing, and the 8:2 and 10:2 FTOH-sulfates were frequently detected between 6 hr and 2 days postdosing. After monoPAP IV dosing, we detected 4:2 FTCA, 4:2 FTUCA, and 6:2 FTCA 2 hr postdosing; 6:2 FTUCA and 5:3 FTCA were observed in the 2, 4, and 6-hr time points. We also detected 4:2 FTOH-sulfate and 4:2 FTOH-glucuronide in the blood 2 hr after dosing.

After diPAP dosing, we detected metabolic intermediates and phase II metabolites only sporadically. After diPAP oral gavage, 10:2 FTCA was present in the blood at the 6-hr time point. We also observed 4:2 FTCA, 4:2 FTUCA, 6:2 FTCA, 6:2 FTUCA, and 8:2 FTCA 6 hr postdosing, but in only one animal. The only phase II metabolite observed was 10:2 FTOH-sulfate at 1 and 2 days after dosing. After IV diPAP dosing, we observed 5:3 FTCA in the samples from 2 hr to 2 days postdosing and 10:2 FTCA only in the sample collected 2 days after dosing. We also detected 4:2 FTOH-sulfate and 4:2 FTOH-glucuronide in the blood 2 hr postdosing. No monoPAPs were observed in the blood of any diPAP-dosed animal.

## Discussion

Migration from paper packaging into food is likely the major route of human PAP exposure. Therefore, understanding bioavailability is important to properly characterize exposure. With observed bioavailabilities of 190%, 74%, and 5% for 4:2, 6:2, and 8:2 diPAP congeners, respectively, aside from potential overestimation, it is clear that in the present study the diPAP congeners were absorbed from the gut contents into the bloodstream. There is also a clear decreasing trend in bioavailability as chain length increases from C4 to C8 perfluorinated carbons.

Telomerization inherently produces a mixture of perfluoroalkyl chain lengths (Kissa 2002), and in the manufacture of fluorotelomer surfactants this process is optimized to produce predominantly the perfluorohexyl chain length (Dupont 2002). We previously found the diPAP congener profile in human sera to be dominated by 6:2 diPAP (D’eon et al. 2009). Increased exposure to the perfluorohexyl chain length, combined with increased bioavailability of the shorter chain lengths, is consistent with the higher concentrations of 6:2 diPAP observed in human sera.

Interest in human diPAP exposure is related both to the diPAPs themselves and to their potential contribution to human PFCA contamination. We observed PFCA biotransformation products in the blood of the diPAP-dosed animals, which are plotted together with their expected diPAP parent in Figure 1. Despite the observation of relatively high PFCA concentrations, we detected metabolic intermediates only intermittently [see Supplemental Material (doi:10.1289/ehp.1002409)]. This result was surprising because it contrasts the high concentrations of 8:2 FTCA observed after 8:2 FTOH gavage in the rat (Fasano et al. 2006). FTOHs are the direct product of diPAP hydrolysis [as shown in Figure 2B; (D’eon and Mabury 2007; Lee et al. 2010)]. Disparity between concentrations of metabolic intermediates observed after diPAP dosing, compared with FTOH dosing, demonstrates that diPAP exposure does not simply mimic FTOH exposure. The low concentrations of metabolic intermediates observed after diPAP exposure may explain why, in contrast to the frequent detection of sulfonamide oxidation products (Calafat et al. 2007; Haug et al. 2009; Olsen et al. 2008), intermediates in the biotransformation of fluorotelomer materials have never been reported in human sera.

Contrary to the observations after diPAP exposure, we did not observe monoPAPs in the blood of the monoPAP-dosed animals. However, we observed 6:2, 8:2, and 10:2 monoPAPs in feces samples collected in the first 24 hr after administration of the monoPAP gavage dose. Because we did not detect monoPAPs in the feces after IV dosing, the concentrations observed after oral gavage presumably result from residual dosing material. The mass of monoPAPs recovered in the feces after oral gavage could account for < 1% of the administered gavage dose, indicating that the monoPAPs did not pass through the gut unaltered but were either absorbed from the gut or biotransformed within the gut contents. It is difficult to delineate between these two possibilities; however, there is literature precedent for enzyme-mediated hydrolysis within the gut contents, because this has been observed in biotransformation studies of 8:2 fluorotelomer acrylate in rainbow trout (Butt et al. 2010).

Despite the lack of monoPAPs observed in the blood, we consistently detected PFCA biotransformation products in the monoPAP-dosed animals (Table 1). We also detected relatively high concentrations of several metabolic intermediates, a result that is similar to previous studies involving FTOH exposure in the rat (Fasano et al. 2006). This consistency between monoPAP and FTOH exposure supports the hypothesis that monoPAP phosphate ester hydrolysis occurs within the gut contents, followed by FTOH uptake and further oxidation, because this route of exposure would essentially mimic direct FTOH exposure.

Differences between diPAP and monoPAP exposure were not limited to relative concentrations of metabolic intermediates. Elimination kinetics of the short-chain PFCA products in the blood [perfluorobutanoic acid (PFBA) and PFHxA] were significantly slower after diPAP dose administration than after monoPAP (*t*-test, *p* < 0.01; Table 1). After diPAP IV dosing, we observed serum elimination half-lives of 3.3 ± 1.2 days and 1.8 ± 0.5 days for PFBA and PFHxA, respectively, whereas after monoPAP IV dosing the observed half-lives were 0.53 ± 0.15 days and 0.23 ± 0.23 days, respectively. We observed a similarly long half-life for PFHxA after diPAP oral gavage (4.2 ± 1.3 days). PFBA and PFHxA have serum half-lives on the order of hours in male rats (Chang et al. 2008; Ohmori et al. 2003); the longer serum half-lives we observed after diPAP dose administration suggest a continual source of these compounds to the animal after the initial bolus dose. This observation is consistent with the gradual biotransformation of diPAP congeners present within the body. Conversely, similarity between PFBA and PFHxA elimination half-lives in the literature (Chang et al. 2008; Ohmori et al. 2003) and those observed after bolus monoPAP exposure supports the hypothesis that most monoPAP biotransformation occurred at the time of dose administration.

If most of the observed diPAP biotransformation occurred within the body, then we can potentially use diPAP concentrations in the blood to predict the resulting PFCA burden. Percent biotransformation will be predicted for the diPAP congeners using the AUC for both the parent diPAP and product PFCA after either IV or oral gavage dose administration using Equation 2:


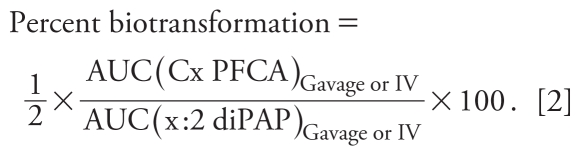


Because this calculation involves conversion between two chemical species, it was performed using blood concentrations expressed in moles as opposed to mass [see Supplemental Material (doi:10.1289/ehp.1002409)]. These biotransformation yields should be considered conservative because they assume the production of 2 mol PFCA from 1 mol diPAP.

Applying this definition of percent biotransformation to the diPAP experiments resulted in < 1% biotransformation for 4:2 diPAP, with this increasing to about 1% for 6:2 diPAP and to about 10% for 8:2 and 10:2 diPAP (Table 1). These percentages do not represent ratios of parent to product concentrations observed in the blood. Instead, they describe the cumulative PFCA burden imparted to the animal from the cumulative diPAP exposure observed in the blood. These predictions are species specific because they are heavily dependent on the pharmacokinetics of both parent and product. Pharmacokinetic parameters for fluorinated acids are not easily scaled between species (Andersen et al. 2008), so caution must be used when comparing predictions made in the rat with situations in other species, including humans.

**Figure 3:**
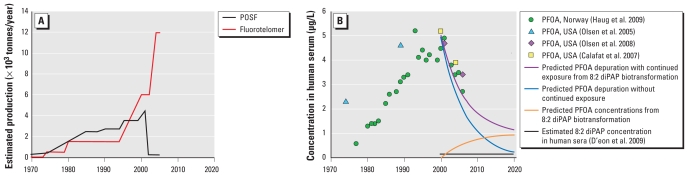
Estimated production volumes (*A*; data from Dupont 2002, 2005; Vestergren and Cousins 2009) and PFOA concentrations observed in human serum (*B*; data from Calafat et al. 2007; Haug et al 2009; Olsen et al. 2005, 2008) for 1970–2020. In *B*, data are plotted with the estimated 8:2 diPAP concentration in human sera [assumed to be constant (0.15 μg/L) from 2000 to 2020], predicted PFOA concentrations in human sera from 8:2 diPAP biotransformation, predicted PFOA depuration without continued exposure, and predicted PFOA depuration with continued exposure from 8:2 diPAP biotransformation alone.

Comparison between contaminant temporal trends in human sera with chemical production can delineate between exposures to current-use materials and legacy chemical use. For both PFOS and PFOA, a concurrent decline in the levels of these contaminants in human sera has been reported with the phase-out of the POSF-based materials (PFOA temporal trend shown in Figure 3B) (Calafat et al. 2007; Haug et al. 2009; Olsen et al. 2008). This trend suggests that POSF production was intimately linked to human PFOS and PFOA exposure. The correlation between POSF production and the PFOS found in human sera is clear, because PFOS can be produced from the biotransformation of the sulfonamide commercial materials (Xu et al. 2004), and sulfonamide oxidation products have been observed in human sera (Calafat et al. 2007; Haug et al. 2009; Olsen et al. 2008). The correlation between POSF production and human PFOA contamination is not as clear. The issue is further complicated by the observation that the decrease in PFOS concentrations was greater than that of PFOA (Calafat et al. 2007; Haug et al. 2009; Olsen et al. 2008), despite experimental data indicating faster human serum elimination kinetics for PFOA than for PFOS (Olsen et al. 2007). The best explanation for this phenomenon is that the observed PFOA elimination kinetics are offset by continued PFOA exposure. The same temporal trends also indicate continued exposure to PFNA, PFDA, and PFUnA (Calafat et al. 2007; Haug et al. 2009; Olsen et al. 2008). Although it is difficult to know whether changes in industrial practice by other fluorochemical producers may have also contributed to human PFOA exposure, the temporal trends in human sera are consistent with POSF-based materials being a significant source of human exposure to PFOS and PFOA until 2000. After 2000, fluorotelomer production continued to increase (Figure 3A), and exposure to fluorotelomer-based materials may explain continued exposure to PFOA, together with increasing exposure to the longer chain PFCAs, such as PFNA, PFDA, and PFUnA.

A major limitation to understanding the connection between human fluorotelomer exposure and PFCA contamination is the ability to constrain PFCA production from fluorotelomer biotransformation. A simple calculation using the biotransformation yields determined here may highlight the potential for fluorotelomer biotransformation to contribute to PFCA contamination. Human serum elimination of PFOA can be predicted using the arithmetic human serum half-life of 4.5 years (Olsen et al. 2007). In Figure 3B, the blue line indicates predicted elimination of PFOA, starting at 5 μg/L in the year 2000, without continued exposure. This prediction estimates PFOA concentrations slightly below the empirical observations. To predict PFOA exposure from 8:2 diPAP biotransformation, we assumed a constant concentration of 0.15 μg/L 8:2 diPAP in human serum from 2000 to 2020 (Figure 3B, black line). This serum concentration was based on 8:2 diPAP levels observed in human sera from the United States in 2008 (D’eon et al. 2009). We then estimated PFOA production from 8:2 diPAP biotransformation by quantizing 8:2 diPAP exposure to 1-month intervals and calculating the expected increase in PFOA sera concentrations using a 10% biotransformation yield [Figure 3B, orange line; for details, see Supplemental Material (doi:10.1289/ehp.1002409)]. Combining predicted PFOA depuration (Figure 3B, blue line) with continued PFOA exposure from 8:2 diPAP biotransformation (Figure 3B, orange line) produces the purple line in Figure 3B, which better predicts the temporal trend of PFOA in human sera. This simple calculation demonstrates the ability of relatively low-level fluorotelomer exposure to produce PFCA burdens similar to those currently observed in human sera.

## Conclusion

Despite the ubiquity of human PFCA and PFSA contamination, major sources of this exposure are not fully appreciated. Attempts to model human exposure have generally focused on the PFSAs and PFCAs present in food items, household dust, and drinking water. These studies have consistently identified food as the major exposure pathway of those investigated (Fromme et al. 2007, 2009; Kärrman et al. 2009; Trudel et al. 2008; Vestergren et al. 2008). PFCA and PFSA contamination of Canadian foodstuffs was unchanged between 1998 and 2004 (Ostertag et al. 2009). Given the marked changes in fluorochemical production during this time period (Figure 3A), this suggests that the contamination of food items results from legacy environmental contamination (Vestergren and Cousins 2009). Consequently, food-borne exposure cannot explain the decrease in PFOS and PFOA levels observed in human serum in North America and Europe that began around the year 2000 (Calafat et al. 2007; Haug et al. 2009; Olsen et al. 2008), in concert with changes in POSF production (3M 2000). Commercial fluorochemical materials and formulations likely do mirror changes in production and market share. As a result, human exposure to commercial materials may explain the concerted changes observed in human contamination with changes in fluorochemical production.

Some attempts have been made to model human PFCA and PFSA exposure via the biotransformation of fluorotelomer and sulfonamide materials and have found this exposure source to be negligible (Fromme et al. 2009; Vestergren et al. 2008). However, these modeling studies are premature because they are limited by a paucity of data regarding concentrations in exposure media as well as a lack of agreement regarding biotransformation yields. In addition, both studies considered human exposure only to residual FTOHs and sulfonamides, and not exposure to the actual commercial materials, which represent the largest potential source of exposure. Using the diPAP commercial surfactants as an example, the high concentrations observed in paper fiber samples (≤ 1,400 ng/g) and wastewater treatment plant sludge (≤ 860 ng/g) indicate that these fluorochemicals are likely present at high concentrations in the indoor environment (D’eon et al. 2009). In migration tests, Begley et al. (2008) observed migration of about 0.5 μg 8:2 diPAP/g butter, implying exposure to about 6 μg 8:2 diPAP for every tablespoon (15 mL) of butter consumed after contact with PAP-treated paper. These exposure estimates demonstrate that exposure to commercial fluorochemicals cannot be ignored and must be better characterized to properly understand human PFCA contamination.

The present study was built on the implicit notion of Hagen et al. (1981) that human PFCA contamination results from the biotransformation of fluorotelomer-based materials. The calculations shown in Figure 3B oversimplify the issue of human PFCA exposure. Nevertheless, they demonstrate that the low-level diPAP contamination observed in human sera could result in significant PFCA exposure, especially for PFCA congeners with human half-lives on the order of years (≥ C8) (Olsen et al. 2007). Exposure to PAPs may be a significant source of PFCA exposure, the degree to which is difficult to assess without proprietary data regarding the production and use of different fluorinated materials. It should also be noted that exposure to fluorotelomer-based materials is not limited to PAPs and could occur via other pathways such as inhalation of volatile precursors (Shoeib et al. 2005; Stock et al. 2004) or ingestion of other fluorotelomer-based surfactants (Begley et al. 2008; Fromme et al. 2009). Overall, diPAP bioavailability and PFCA biotransformation products observed in the present study demonstrate that exposure to PAPs could be a significant contributor to the PFCA burden currently observed in humans.

## Figures and Tables

**Table 1:** Pharmacokinetic parameters for the diPAPs and PFCA biotransformation products determined from analyte concentrations in whole blood.

		Gavage dose				IV dose			
Acronym	Bioavailability (%)	Biotransformation^a^ (%)	Half-life (days)	*T*_max_	*C*_max_ (ng/g)	Biotransformation^a^ (%)	Half-life (days)	*T*_max_	*C*_max_ (ng/g)
diPAP dose									

4:2 diPAP	190	0.005	2.0 ± 0.1	6 hr	39,000 ± 4,000	0.5	1.6 ± 0.1	2 hr	14,000 ± 1,000
6:2 diPAP	74	0.6	3.9 ± 0.7	4 hr	1,500 ± 500	1	2.1 ± 0.3	2 hr	2,200 ± 200
8:2 diPAP	5	9	2.4 ± 0.3	1 day	400 ± 60	9	4.8 ± 1.0	1 day	490 ± 120
10:2 diPAP	NA^b^	NA^b^	NA^b^	NA	ND	8	3.3 ± 0.4	1 day	480 ± 110
PFBA	—	—	NA^c^	1 day	3.8 ± 1.0	—	3.3 ± 1.2	1 day	20 ± 5
PFPeA	—	—	—	NA	ND	—	—	2 hr	7.1 ± 0.6
PFHxA	—	—	4.2 ± 1.3	4 hr	10 ± 1.2	—	1.8 ± 0.51	1 day	(3.0 ± 1.0)^d^
PFHpA	—	—	—	1 day	2.6 ± 0.5	—	—	4 hr	7.3 ± 0.6
PFOA	—	—	11 ± 2	4 days	7.2 ± 1.7	—	23 ± 17	2 days	9.3 ± 2.4
PFNA	—	—	—	8 days	6.4 ± 5.2	—	—	15 days	(4.1 ± 3.3)
PFDA	—	—	11 ± 2	1 day	3.6 ± 0.6	—	10 ± 2	2 days	6.2 ± 1.2
PFUnA	—	—	—	NA	ND	—	—	NA	ND

monoPAP dose									

PFBA	—	—	NA^c^	1 day	9.7 ± 1.9	—	0.53 ± 0.15	6 hr	7.3 ± 1.3
PFPeA	—	—	—	NA	ND	—	—	6 hr	2.1 ± 0.8
PFHxA	—	—	NA^3^	4 hr	7.4 ± 1.5	—	0.23 ± 0.23	4 hr	5.6 ± 1.3
PFHpA	—	—	—	1 day	9.0 ± 2.2	—	—	1 day	2.3 ± 0.7
PFOA	—	—	10 ± 2	1 day	28 ± 8	—	12 ± 6	4 days	8.5 ± 1.4
PFNA	—	—	—	1 day	(1.5 ± 0.09)	—	—	15 days	2.3 ± 0.4
PFDA	—	—	12 ± 8	2 days	(1.1 ± 0.27)	—	13 ± 9	15 days	0.93 ± 0.20
PFUnA	—	—	—	NA	ND	—	—	NA	ND

Abbreviations: —, not relevant; *C*_max_, maximum concentration; NA, not available; ND, not detected; PFHpA, perfluoroheptanoic acid; PFPeA, perfluoropentanoic acid; *T*_max_, time of maximal concentration. monoPAP is not included because it was not found in any blood sample. Values < LOD were assigned a value of zero; values < LOQ but > LOD are shown in parentheses. For full data sets, including concentrations of metabolic intermediates and phase II metabolites, see Supplemental Material, Tables 13–24 (doi:10.1289/ehp.1002409).

a Biotransformation was calculated using Equation 2.

b 10:2 diPAP was not observed in the blood after diPAP gavage dosing, so no parameters that require these concentrations could be calculated.

c Because the excretion phase was not properly captured, no half-life could be calculated for PFBA after diPAP gavage dosing, or for PFBA and PFHxA after monoPAP gavage dosing.

d The first three PFHxA time points after diPAP intravenous dosing were omitted because of inadvertent PFHxA contamination of the dose, as described in the text.
